# Comparison of the relative impacts of acute consumption of an inulin-enriched diet, milk kefir or a commercial probiotic product on the human gut microbiome and metabolome

**DOI:** 10.1038/s41538-023-00216-z

**Published:** 2023-08-16

**Authors:** Liam H. Walsh, Aaron M. Walsh, Isabel Garcia-Perez, Fiona Crispie, Adele Costabile, Richard Ellis, Jim Finlayson, Laura A. Finnegan, Marcus J. Claesson, Elaine Holmes, Paul D. Cotter

**Affiliations:** 1 Teagasc Food Research Centre, Moorepark, Fermoy, Co, Cork, Ireland; 2 School of Microbiology Department, University College Cork, Co, Cork, Ireland; 3 APC Microbiome Ireland, University College Cork, Co, Cork, Ireland; 4 Section of Biomolecular Medicine, Division of Computational Systems Medicine, Imperial College London, London, UK; 5 School of Life and Health Sciences, University of Roehampton London, London, UK; 6 Surveillance and Laboratory Services Department, APHA, Addlestone, UK; 7 NHS Highland, Highland Clinical Research Facility, University of the Highlands & Islands, Centre for Health Science, Inverness, UK; 8 VistaMilk SFI Research Centre, Teagasc, Moorepark, Fermoy, Co, Cork, Ireland

**Keywords:** Food microbiology, Metagenomics

## Abstract

It has been established that the human gut microbiota is central to health, and, consequently, there has been a growing desire to positively modulate its composition and/or function through, for example, the use of fermented foods, prebiotics or probiotics. Here, we compare the relative impact of the daily consumption of an inulin-enriched diet (*n* = 10), a commercial probiotic-containing fermented milk product (FMP) (*n* = 10), or a traditional kefir FMP (*n* = 9), over a 28-day period on the gut microbiome and urine metabolome of healthy human adults. None of the treatments resulted in significant changes to clinical parameters or biomarkers tested. However, shotgun metagenomic analysis revealed that kefir consumption resulted in a significant change in taxonomy, in the form of an increased abundance of the sub-dominant FMP-associated species *Lactococcus raffinolactis*, which further corresponded to shifts in the urine metabolome. Overall, our results indicated that daily consumption of a single portion of kefir alone resulted in detectable changes to the gut microbiota and metabolome of consumers.

## Introduction

It is becoming increasingly apparent that the microorganisms in the human gut are pivotal to many aspects of our health. Disturbance of the gut microbiota has been linked to a variety of diseases, including colon cancer, diabetes, inflammatory bowel disease, and obesity^1^. Consequently, gut microbiota has emerged as a potential target for the prevention or treatment of such diseases. Targeted manipulation of the gut microbiota can be achieved by dietary intervention, including through the use of fermented foods, probiotics or prebiotics^2^.

A probiotic is a live microorganism that exerts health benefits upon the host when it is ingested in sufficient quantities^3^. Many probiotic strains are consumed as constituents of yogurt/fermented milk-type drinks or as supplements in the form of capsules. To access the lower gastrointestinal tract, probiotics must first survive transit through the acidic upper gastrointestinal tract. Some probiotic strains can subsequently colonise the gut by adhering to the intestinal epithelial cells^4^. A number of potential mechanisms can contribute to the health benefits of specific strains. These include pathogen inhibition via bacteriocin production or mucosal competitive exclusion, and immunomodulation^5^. Human studies have demonstrated that probiotic strains, such as *Bifidobacterium infantis* 35624, *Lacticaseibacillus casei* DN-114001, and *Lacticaseibacillus casei* Shirota, confer health benefits, including alleviation of the symptoms of irritable bowel syndrome, prevention of antibiotic-associated diarrhoea, or protection against infection^6–8^.

Health benefits have also been attributed to several traditional fermented foods and the microbes therein^9^. Particular examples of kefir, a traditional fermented milk product (FMP), have been linked to health benefits, including anti-cholesterolemic, anti-inflammatory, and anti-pathogenic effects^10–12^, and several investigations indicate that specific microorganisms in kefir contribute to these effects^13–15^. Despite this promise, it should be noted there has been a relative lack of research into the effects of kefir on human health, with most investigations relying on in vitro or animal models^16^.

Prebiotics can also beneficially influence the gut microbiota. Prebiotics are non-digestible oligosaccharides that stimulate the growth of health-promoting commensal microorganisms in the gut^17^. The most frequently studied prebiotics are fructooligosaccharides, galactooligosaccharides, and inulin^18^. Prebiotics naturally occur in various foods, including artichokes, chicory and wheat, but can also be provided in the form of food supplements^19^. Human studies have demonstrated that prebiotics can alter the gut microbiome, with many studies reporting an increase in *Bifiodobacterium* and or members of the former genus *Lactobacillus*^20^, as well as confering various health benefits, including improved satiety, lowered insulin concentrations, and reduced infection^21–23^.

Metagenomic and metabolomic-sequencing efforts are beginning to improve our understanding of the effects of probiotics, prebiotics and fermented foods on the human gut microbiota and metabolome^24,25^. However, the majority of studies have been directed at preventing or treating the symptoms of specific diseases^26^ and/or evaluating the impact of consumption of high quantities of a probiotic(s), prebiotic(s) and/or fermented food(s)^27^. Few studies to date have analysed the relative impact of supplementing diets with moderate portions of one of these products on the microbiota and metabolome of healthy participants^28^.

In the present study, we use metagenomic sequencing and metabolomics to identify changes in the human gut microbiota and metabolome of healthy participants following daily consumption, over 28 days, of either a single portion of a commercial FMP containing the probiotic *L. casei*, a traditional fermented milk beverage kefir or a diet enriched with the prebiotic inulin.

## Results

### Clinical outcomes

For this study, we investigated clinical and anthropomorphic parameters, the composition and functional potential of the gut microbiota and metabolome profile of healthy participants following daily consumption (28 days) of either a commercial probiotic-containing FMP, a traditional kefir FMP or a diet enriched with the prebiotic inulin. Several parameters were measured before and after treatment and none of the treatments resulted in significant changes to the clinical parameters or biomarkers tested, i.e., percentage of body fat, anxiety, and abdominal symptoms (Supplementary Table 1).

### Compositional analysis reveals an increased abundance of *Lactococcus raffinolactis* after kefir consumption

**Fig. 1:**
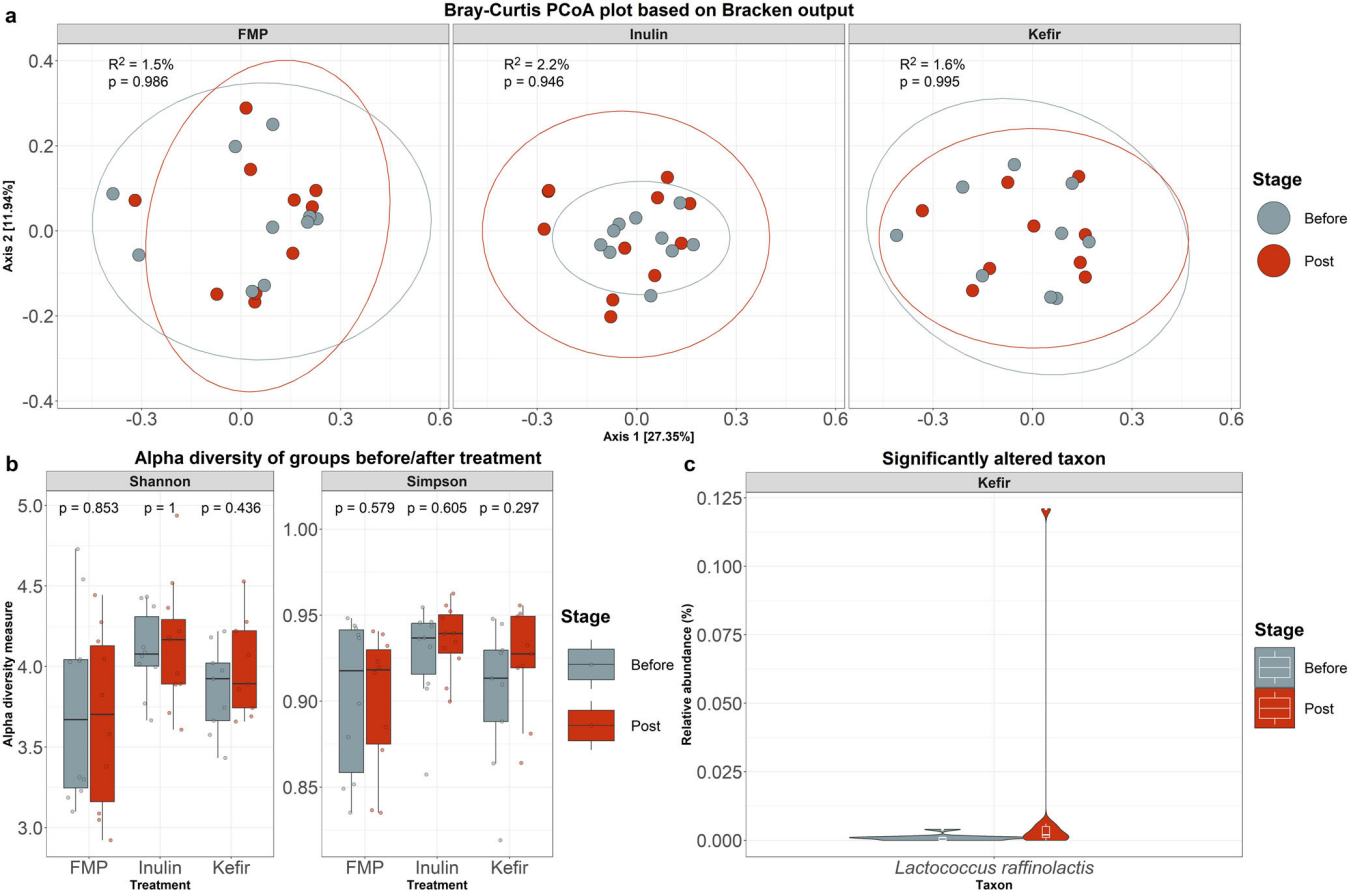
Changes in microbiome diversity following FMP, Inulin and Kefir consumption. **a** Principal coordinate analysis (PCoA) of beta diversity by intervention, measured by Bray–Curtis dissimilarity and calculated for species-level composition. **b** Changes in alpha diversity post interventions, values calculated using the Shannon and Simpson diversity metrics. *P*-values represent the results of separate Wilcoxon signed-rank tests. **c** Changes in the relative abundance of *Lactococcus raffinolactis* post kefir consumption. The bounds, whiskers and percentile of each box plot represented maximum, 75th percentile, median, 25th percentile and minimum from the top to the bottom respectively.

**Fig. 2:**
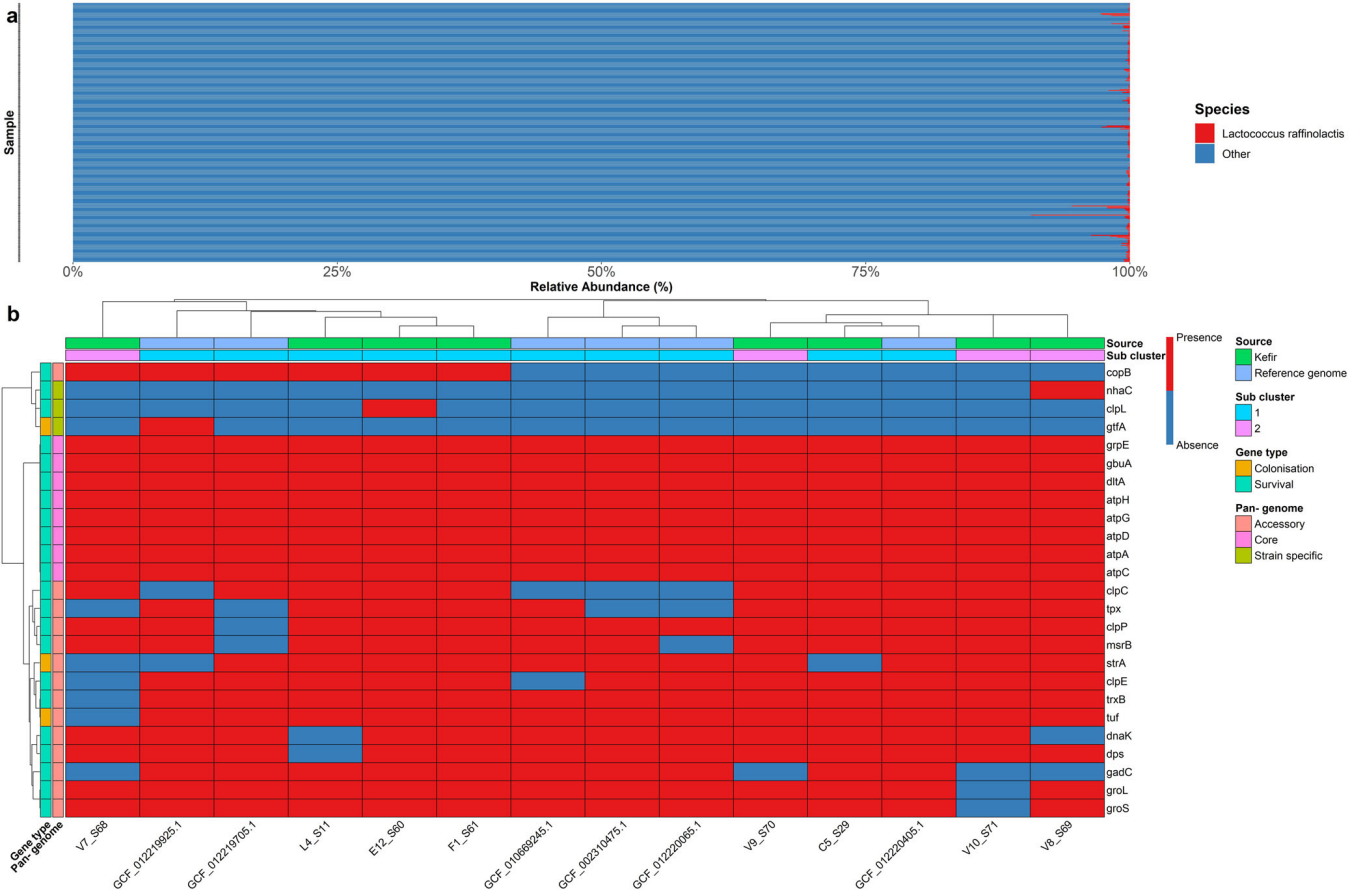
Prevalence of *Lc. raffinolactis* strains within the milk kefir microbiome and host adaptability genes within the pan- genome of *Lc. raffinolactis*. **a** Prevalence of *Lc. raffinolactis* across 256 kefir metagenomes generated by Walsh et al. and assessed using Kraken2. **b** Prevalence of genes with potential applications in host adaptability (*Y*-axis) across references of species *Lc. raffinolactis* (*X*-axis). The colour of each cell of the heatmap represents the presence (red) or absence (blue) of host adaptability genes. Row side annotations include gene classifications separated into “Colonisation”, and “Survival”, summary descriptions of the pan-genomes of *Lc. raffinolactis*, separated into “Core”, “Accessory” and “Strain specific” based on the prevalence of genes across reference genomes. Column side annotations represent the source data and sub-cluster of the (Meta) genome.

Shotgun metagenomics was used to compare the gut microbial diversity and taxonomic abundance for all participants before versus after treatment, using the taxonomic profiling tool Kraken 2. When considering microbial diversity, no significant changes were observed, as determined using statistical analysis and MDS with no perceivable shift in the composition of the gut following inulin (PERMANOVA: *p* = 0.94, *R*^2^ = 2.2%), kefir (PERMANOVA: *p* = 0.99, *R*^2^ = 1.6%), or commercial FMP (PERMANOVA: *p* = 0.99, *R*^2^ = 1.5%) (Fig. 1a, b), Shotgun metagenomics was used to determine which of the species detected in the gut microbiome of participants were differentially abundant after consumption of either inulin, kefir or a commercial FMP. Species were selected based on the reported microbial compositions of the probiotic (https://www.hcp.yakult.co.uk/) and kefir^29^ and through a literature search for inulin, for example, inulin has previously been shown to modulate the genera *Anaerostipes*, *Bifidobacterium*, and *Bilophila*^30^. No differentially abundant species were observed in the commercial FMP or inulin group. However, in the kefir group we observed the increased relative abundance of *Lactococcus raffinolactis* in 4 of the 10 participants, which was below detectable levels prior to kefir consumption (Fig. 1c). The presence of *Lc. raffinolactis* in these samples was confirmed using the alignment-based approach raspir (rare species identifier)^31^. *Lc. raffinolactis* is a prevalent microorganism within the milk kefir microbiome, taxonomic profiling of 256 kefir milk metagenomes revealed its presence in 169 of those metagenomes at a relative abundance of 0.1–9%. (Fig. 2a).

### Comparative genomics reveals a number of host adaptability features within the pan-genome of *Lc. raffinolactis*

Reads mapped to the *Lc. raffinolactis* genome during the rapsir methodology was further inspected for annotated genes whereby the survival genes *dltA*, *clpE*, *copB_1* and *tuf* were identified from gut metagenomics data post kefir consumption. Given the recovery of survival genes from gut metagenomics data post kefir consumption, a comparative genomics approach was employed to uncover further, host adaptability features across the *Lc*. *raffinolactis* species (Fig. 2b)^32^. The pan-genome of *Lc. raffinolactis* contained 22 survival genes and suggested resistance to multiple stress conditions. The pan-genome included genes encoding stress-related proteins such as the molecular chaperones DnaK, GroES and GroEL, heat shock protein such as GrpE and oxidative stress-resistant genes such as thiol peroxidase and thioredoxin reductase^33^. Furthermore, transport system-associated genes/annotations such as *gbuA* were identified. *gbuA* encodes a component of the choline ABC transport system with applications in osmoregulation^34,35^ (Fig. 2b). Three genes associated with adhesion-related functions were identified in the pan-genome of *Lc. raffinolactis* including cell-surface-related proteins, such as elongation factor Tu (*tuf*), and sortase A-dependent enzymes (StrA) (Fig. 2b), which is known to affect the mucin binding ability of microorganisms^36^. 5 Glycoside hydrolase family (GH) enzymes (GH36-EC 3.2.1.22, GH42-EC 3.2.1.23, GH109-EC 3.2.1.49, GH29-EC 3.2.1.51 and GH20-EC 3.2.1.52) were recovered with potential applications in mucin cleavage. Additionally, the GH29 family enzymes (alpha-l-fucosidase-EC 3.2.1.51, alpha-1,3/1,4-l-fucosidase-EC 3.2.1.111) suggested strains could remove Fuc moieties contained in intestinal mucin glycans^37–39^. Supportive annotations for the production of the metabolites lactate and acetate and a number of genes linked to GABA production were widely found across the *Lc. raffinolactis* references. Specifically, the identified genes included the *puuD* gene encoding for a γ-Glu-GABA hydrolase, which plays a role in the Puu pathway by converting γ-Glu-GABA into GABA^40^ and the genes *gadB* and *gadC* encoding for glutamate decarboxylases (GadB) and Glu/GABA antiporter, respectively. The glutamic acid decarboxylase (GAD) system provides a full mechanism by which *Lc. raffinolactis* can produce GABA^41^. All genomes encoded for β-galactosidase activity (EC 3.2.1.23), which could aid the digestion of lactose^42^.

Within the genus *Lactococcus, Lc. lactis* strains are generally recognised as safe (GRAS) by the Food and Drug Administration (FDA)^43^. *Lc. lactis* together with *Lc. raffinolactis* are listed among the inventory of microbial food cultures (MFC) of fermented food products as species with demonstrated safety^44^. Comparative genomic analysis of *Lc. raffinolactis* revealed no functional genes encoding enterotoxins, transferable antibiotic resistance, and an absence of d-lactate dehydrogenase (d-LDH) (EC 1.1.1.28) across strains that harboured only l-LDH (EC 1.1.1.27). d-LDH represents a safety concern as d-lactate produced by commensals may induce d-lactate acidosis^45^. All *Lc. raffinolactis* genomes also contained the cold shock gene *cspL*, which may support the survival of cold stress conditions imposed during cold temperature storage^46^.

### Kefir consumption results in modest changes to urinal metabolites but not functional potential

HUMAnN3 was used to measure the abundance of pathways encoded within the faecal microbiome. MDS coupled with statistical analysis indicated that, overall, there was no perceivable shift in the functional potential of the microbiome following the consumption of inulin (PERMANOVA: *p* = 0.95, *R*^2^ = 0.2%), kefir (PERMANOVA: *p* = 0.75, *R*^2^ = 0.8%), or commercial FMP (PERMANOVA: *p* = 0.573, *R*^2^ = 2.1%) (Supplementary Fig. 1).

Three MCCV–PLS-DA models were built to investigate the impact of kefir, yogurt and inulin interventions on urinary metabolites. No significant differences were found following consumption of commercial inulin (*R*^2^*Y* = 0.98, *Q*^2^*Y* = −0.28), FMP (*R*^2^*Y* = 0.96, *Q*^2^*Y* = −0.8) or kefir (*R*^2^*Y* = 0.99, *Q*^2^*Y* = 0.57).

**Fig. 3:**
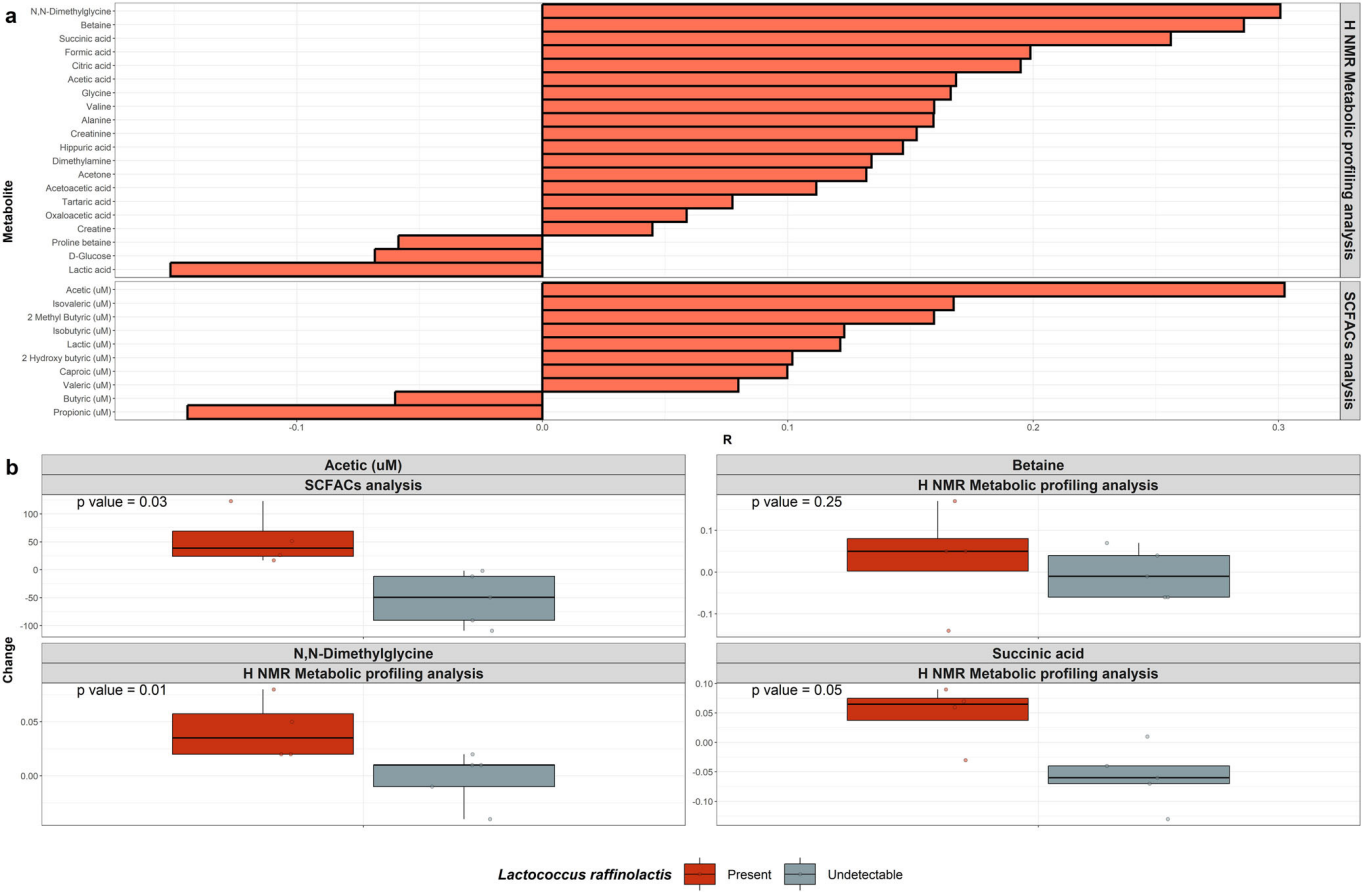
The presence of *Lc. raffinolactis* coincides with statistically significant shifts in the metabolome of the kefir cohort. **a** Correlations between the concentrations of metabolites and the relative abundance of *Lc. raffinolactis.* **b** Changes in the quantities of the positively correlated metabolites Acetic, Betaine, N,N-Dimethylglycine and Succinic acid, post kefir treatment, separated according to the undetectable (grey) or present (red) status of *Lc. raffinolactis*. *P*-values reflect the output of separate Wilcoxon signed ranked tests assessing the change in metabolites between participants of the kefir cohort. The bounds, whiskers and percentile of each box plot represented maximum, 75th percentile, median, 25th percentile and minimum from the top to the bottom, respectively.

The relative abundance of *Lc. raffinolactis* was positively correlated with the concentrations of acetic, N,N-dimethylglycine, betaine and succinic acid (Fig. 3a). However, none of these correlations were significant following *p*-value adjustment. While none of the metabolites were significantly altered when considering the kefir cohort in its entirety, we noted the metabolites acetic (Wilcoxon: *p* = 0.03), N,N-dimethylglycine (Wilcoxon: *p* = 0.01) and succinic acid (Wilcoxon: *p* = 0.05) were statistically increased post kefir consumption in participants with increased relative abundance of *Lc. raffinolactis*, compared with the remaining participants of the kefir cohort (Fig. 3b). Furthermore, the examined *Lc. raffinolactis* genomes contained complete annotations for acetic acid production and supporting annotations for succinic acid production through the TCA cycle and N,N-dimethylglycine through the glycine betaine/proline transport system. Furthermore, *Lc. raffinolactis* genomes have supportive metabolic pathways to produce GABA, which can be metabolised to succinic acid, through the metabolic pathway GABA shunt, which was increased post kefir consumption.

## Discussion

In the present study, we combined whole metagenome shotgun sequencing and metabolomics to investigate the respective effects of a daily portion of an inulin, kefir, and a commercial probiotic drink containing *Lacticaseibacillus casei* on the human gut microbiota. All participants across the three treatment groups were deemed healthy and displayed individualised microbiomes (Fig. 1a). As expected, our findings indicated that none of the treatments caused major changes in the overall human gut microbiota structure. It is important to note that these findings result from a relatively small number of participants. As the effect size is minimal, greater numbers of samples would be required to detect more subtle changes to the faecal microbiome arising as a result of the consumption of these treatments.

Despite limitations in sample number, whole metagenome shotgun sequencing revealed changes in the relative abundance of *Lc. raffinolactis* in 4 out of 10 participants after kefir consumption (Fig. 1). The detection of *Lc. raffinolactis* is of particular interest given its “non-dominant”, status compared to other lactococci in dairy foods^47^ and this study is to our knowledge the first to link the detection of *Lc. raffinolactis* in the gut microbiome to fermented food consumption. As the study did not include a wash-out period, it is not clear if the *Lc. raffinolactis* detected represent transient or mucosa-adherent strains, though the detection of the species in only four participants was notable. A number of publications have reported short-term resilience of gut microbiomes to dietary changes including the incorporation of fermented foods, which may explain why *Lc. raffinolactis* was absent in 5 of the 9 participants in the kefir treatment group^27,48^. Alternatively, other studies have demonstrated that the persistence of potentially colonising microbes is dependent on the initial composition of the microbiota^49^, in which the participants of this study displayed individuality (Fig. 1).

Comparative analysis was employed to explore the genomic potential of strains of *Lc. raffinolactis* as a potentially colonising species. A total of 25 genes associated with host adaptability were identified to be contained in at least one of the *Lc. raffinolactis* strains. Specifically 22 survival genes and 3 colonisation genes were recovered across the strains (Fig. 2). The recovery of such genes primarily provides an insight into the possible defence mechanisms employed by *Lc. raffinolactis* to counteract intracellular damage or to enhance the robustness of the cell to withstand the challenging conditions of the gastrointestinal tract and other environmental conditions. CAZymes and peptidases were identified that further supported a colonisation effect and suggested a potential interaction between strains and the colonic mucus, for example, the enzyme families GH109 and GH42 associated with mucin cleavage functionality^50–52^ and the peptidase family Sortase A-C60A associated with mucin binding ability^36^. It should be noted that while surveying and reporting genes associated with host adaptability highlight the potential of *Lc. raffinolactis* as a candidate coloniser, experimental verification is lacking.

Metabolic profiling analysis was used to assess the respective impacts of each treatment on the urine metabolome. No significant changes were observed following either inulin or commercial FMP consumption, but there were significant changes following kefir consumption in a number of these four participants. More specifically, statistically significant changes were observed at the metabolic level in response to consumption of kefir and the presence of *Lc. raffinolactis* with a statistically significant increase in the metabolites acetic acid, N, N-dimethylglycine and succinic acid (Fig. 3b). However, correlation analysis of increased relative abundance of *Lc. raffinolactis* did not display statistically significant linear relationships with the concentrations of any of these metabolites following *p*-value adjustment. Thus, there was no evidence to suggest that *Lc. raffinolactis* had direct effects on the urinal metabolome.

Our findings suggest that kefir consumption in a healthy cohort can have a subtle impact on the urinal metabolome and gut microbiome in a subset of participants. The principle change to the gut microbiome was the detection of *Lc. raffinolactis*. It should be noted that this, and the other interventions, could have more considerably impacted the microbiome of the upper GI tract, like the caecum or the ileum, which would not be detectable in stool samples to the same degree^53^. Regardless, it is unclear if the observed changes are sufficient to confer noticeable health benefits in this already healthy population, especially as we did not find any significant differences in the clinical parameters measured here. It is also important to note that this study used a small sample size of healthy participants and *Lc. raffinolactis* was present in low relative abundance. Due to these limiting factors, the sample size was insufficient to detect functional/metabolic features associated with its presence. Ultimately, this study highlights the need to employ deep metagenomics sequencing in larger cohorts to examine the potentially colonising microbial members of “live foods”, which will likely account for low abundance in the gut^54^, and/or metatranscriptomics to investigate their influence on microbial gene expression in the gut or confirm their viability.

## Methods

### Participant enrolment, intervention design, urine and stool collection

We recruited 29 healthy volunteers, aged from 18 to 65, for this study. Volunteers were members of the public and were enroled from the Centre for Health Science Inverness (https://www.nes.scot.nhs.uk/contact-us/centre-for-health-science-inverness/). Volunteers provided written informed consent to take part in the study. Volunteers had not consumed probiotic-containing products, including commercial probiotic drinks or probiotic supplements, within the 6 weeks preceding the study. They had not taken antibiotics, antacids or proton pump inhibitors within the 2 weeks preceding the study. Volunteers did not have any of the following medical conditions: inflammatory bowel disease (IBD), irritable bowel syndrome (IBS), coeliac disease, food allergies, gastroenteritis (within the 4 weeks preceding the study), heart valve abnormalities, prior rheumatic fever, diabetes or any other immune disorder. Volunteers did not follow any other dietary or lifestyle recommendations during this intervention. After a 7-day run-in period (Day −7 to Day 0), volunteers consumed one of the following treatments daily over a 28-day intervention period (Day 0 to Day 28): 7 g of inulin (*n* = 10), 247 ml of traditional kefir (*n* = 9), and 65 ml of a commercial probiotic (*L. casei* Shirota)-containing dairy beverage (*n* = 10). The inulin group was asked to consume additional portions of foods such as vegetables and fruits that constituted part of their normal diet and contained fibre, which amounted to 7 g of natural inulin. Seven grams of natural inulin were selected based on the GI tolerance^55^, as short- and long-term consumptions of inulin, given at a daily dose containing at least 5 g of inulin, are reported to be well tolerated by healthy subjects^56^. The kefir group was asked to consume a kefir milk product, produced by the inoculation of milk with a kefir grain. Kefir samples were kindly provided by Nourish Kefir (https://www.nourishkefir.co.uk/buy-kefir-here/). Lastly, the probiotic group where asked to consume a commercially available probiotic yoghurt-style drink, containing live cultures of *L. casei* Shirota, Each commercial probiotic contained a minimum of 2 × 10^10^ CFUs, according to the manufacturers (https://www.hcp.yakult.co.uk/). Subjects were controlled weekly via phone calls for possible side effects and compliance in achieving the dietary requirements. Stool and urine samples were collected on Day 0 and Day 28. NHS Ethical Approval was not required as this study involved healthy volunteers only, who provided authorisation for the use of their bodily matter for research purposes in accordance with the NHS research governance systems (https://www.nhsresearchscotland.org.uk/services/research-governance#:~:text=Research%20Governance%20concerns%20setting%20standards,preventing%20poor%20performance%20and%20misconduct). R&D Management Approval was obtained from the NHS Highland R&D Department. The study was conducted according to the Declaration of Helsinki following Good Clinical Practice (GCP).

### Clinical measurements

Several clinical parameters were measured before and after treatment, including height, weight, and body fat. Anxiety was assessed using the GAD-7 questionnaire^57^, while quality of life was assessed using the EQ-5D questionnaire^58^. Abdominal symptoms regarding bloating, flatulence and bowel habit were also recorded. Stool consistency was classified using the Bristol Stool Scale (BSS)^59^.

### Microbial DNA extraction from faeces

Microbial DNA was extracted from 250 mg of faecal samples, using the QIAamp DNA Stool Mini Kit (QIAGEN, UK) according to the protocol described in ref. ^60^.

### High-throughput DNA sequencing

Whole-metagenome shotgun libraries were prepared using the Nextera XT kit in accordance with the Nextera XT DNA Library Preparation Guide from Illumina, with the exception that tagmentation time was increased to 7 min. Samples were sequenced on the Illumina NextSeq 500 in the Teagasc sequencing facility, with a NextSeq 500/550 High Output Reagent Kit v2 (300 cycles), in accordance with standard Illumina sequencing protocols.

### Bioinformatics

Raw whole-metagenome shotgun sequencing reads derived from faecal samples collected in this study and kefir samples generated by Walsh et al.^29^, were quality filtered and trimmed using a combination of Picard Tools v2.18.23 (https://github.com/broadinstitute/picard) and SAMtools v1.10^61^. Kraken2 v 2.1.1^62^ was used to determine the species-level microbial composition of the gut and kefir microbiome. Relative abundance of each species among samples was calculated using Bracken v2.2^63^. To distinguish between false positive and present taxa, the Kraken2 script was performed using the parameter -report-minimizer-data^62^. Identifications displaying a high distinct minimiser count but accounting for <1% relative abundance were confirmed as true positive/negative results using the bioinformatics tool raspir v101 following the methodology outlined in ref. ^31^. Microbial pathway analysis was performed using HUMAnN3 v3.1.1. The pan-genome of *Lactococcus raffinolactis* (*Lc. raffinolactis*) was constructed by Roary v3.13^64^, using complete reference genomes available in NCBI^65^. Kefir-derived metagenome-assembled genomes of the species *Lc. raffinolactis* were acquired from Cotter et al.^29^. Meta(genomes) were clustered using dREP v3.2.0^66^ (Fig. 2). Meta(genome) annotation was performed using DRAM v1.2^67^ and Prokka v1.14^68^. A list of genes associated with survival/colonisation-associated activity was sourced from Mils et al.^69^, Leech et al.^70^ and Kim et al.^71^ and used to examine the host adaptability potential of meta(genomes).

### ^1^H NMR metabolic profiling analysis

Urine samples were stored at −80 °C prior to analysis. Urine samples were thawed at 4 °C and then vortexed and centrifuged at 1600 × *g* for 10 min to remove precipitated proteins and particulates. An aliquot of each urine sample (540 μL) was mixed with 60 μL of phosphate buffer (pH 7.4, 80% H_2_O) containing 1 mM of the internal standard, 3-(trimethylsilyl)-[2,2,3,3,-2H4]-propionic acid (TSP) and 2 mM sodium azide (Na^3^N), as described previously^50^. Urine samples were analysed in 96-well plates containing one quality control (QC) sample every 10 samples. QC samples were prepared by pooling 20 µl volumes of each urine sample. During the analyses, samples were maintained at 4 °C in the autosampler. ^1^H NMR spectroscopy was performed at 300 K on a Bruker 600 MHz spectrometer (Bruker BioSpin, Karlsruhe, Germany) using the following standard one-dimensional pulse sequence with the saturation of the water resonance: RD−gz,1−90°−t−90°−tm−gz,2−90°−ACQ, where RD is the relaxation delay, *t* is a short delay typically of about 4 μs, 90° represents a 90° radio frequency (RF) pulse, tm is the mixing time (10 ms), gz,1 and gz,2 are magnetic field *z*-gradients both applied for 1 ms, and ACQ is the data acquisition period (2.7 s). Water suppression was achieved through continuous wave irradiation at the water resonance frequency using 25 Hz RF strength during RD and also during tm. The receiver gain was set to 90.5 for all experiments. Each urine spectrum was acquired using 4 dummy scans, 32 scans, 64 K time domain points and with a spectral window set of 20 ppm. Prior to the Fourier transformation, the free induction decays were multiplied by an exponential function corresponding to a line broadening of 0.3 Hz.

The ^1^H NMR spectra were digitised over the range of δ10.0 to −0.5 and imported into MATLAB (2014a, Mathworks Inc., USA), and automatically corrected for phase and baseline distortions and referenced to the TSP singlet at δ 0.0 using TopSpin 3.1 software. Spectra were then digitised into 20k data points at a resolution of 0.0005 ppm using an in-house MATLAB R2014a (Mathworks) script. Subsequently, spectral regions corresponding to the internal standard (δ −0.5 to 0.5) and water (δ 4.6–5) peaks were removed. In addition, urea (δ 5.4–6.3) was removed from the urinary spectra. Spectra were normalised using median fold change normalisation to the median spectrum^51^. The resulting ^1^H-NMR spectrum was imported into MATLAB to conduct multivariate statistical analysis. Data were centred and scaled to account for the repeated-measures design and then modelled using partial least squares-discriminant analysis (PLS-DA) with Monte-Carlo cross-validation (MCCV)^52^. The fit and predictability of the models obtained were determined by the *R*-squared and *Q*-squared values, respectively.

### Short-chain fatty acids (SCFAs) analysis

The GC–MS targeted SCFA analysis was conducted on an Agilent 7000C Triple Quadrupole GC/MS System according to a previously published method (24). To interrogate the SCFAs results the non-parametric Wilcoxon’s signed rank test was used to assess pairwise differences.

### Statistics

Statistical analysis was performed in R-3.2.2^72^. The Wilcoxon -signed-rank test was used to identify significant changes in the gut microbiota of faecal samples collected on Day 0 (i.e., before consumption) versus Day 28 (i.e., after consumption) (Fig. 1b). Resulting *p*-values were corrected for multiple comparisons using the Benjamini–Hochberg (BH) method. A result was considered statistically significant if the adjusted p-value was less than 0.1. The significance threshold was relaxed to account for the high number of comparisons. The meta.mds function in the vegan package^73^ was used for multidimensional scaling (MDS) analysis, while the adonis function, also in the vegan package, was used for PERMANOVA analysis (Fig. 1a and Supplementary Fig. 1). Data visualisation of summary statistics generated from the above tests was performed using the ggplot2 package^74^.

### Reporting summary

Further information on research design is available in the Nature Research Reporting Summary linked to this article.

### Supplementary information

Supplementary material

Reporting Summary

## Data Availability

Sequence data has been deposited in the European Nucleotide Archive (ENA) under the project accession number PRJEB26842.

## Code availability

Custom scripts, used for figure generation, metagenomics and statistical analysis and are available at https://github.com/LiamHWalsh/BBC_dietary_intervetion.
