# Supplementary material for: Comparison of the relative impacts of acute consumption of an inulin-enriched diet, milk kefir or a commercial probiotic product on the human gut microbiome and metabolome
Source: NPJ Sci Food. 2023 Aug 16;7:41. doi: 10.1038/s41538-023-00216-z (PMC10432396; doi:10.1038/s41538-023-00216-z)
Supplement: Supplementary file 1 — Supplementary material [file 41538_2023_216_MOESM1_ESM.pdf]

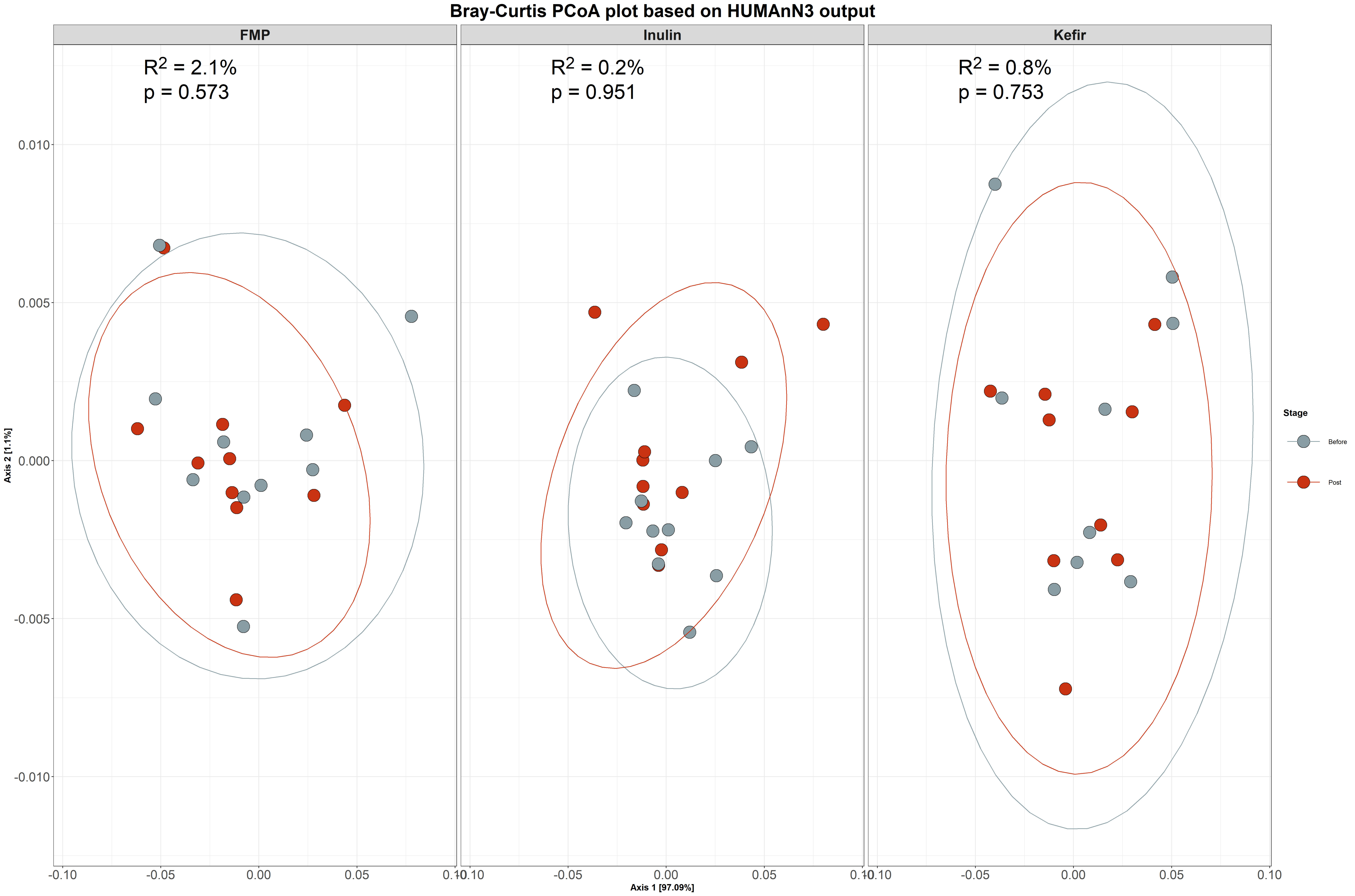

Bray-Curtis PCoA plot based on HUMAnN3 output
FMP
Inulin
Kefir
R2 = 2.1%
p = 0.573
R2 = 0.2%
p = 0.951
R2 = 0.8%
p = 0.753
0.010
0.005
0.000
-0.005
-0.010
Axis 2 [1.1%]
-0.10
-0.05
0.00
0.05
0.10
Axis 1 [97.09%]
Stage
Before
Post

Supplementary figure 1: Changes in metabolic pathway diversity in the gut microbiome following FMP, Inulin and Kefir consumption. Principal Coordinate analysis (PCoA) of beta diversity by intervention measured by Bray-Curtis dissimilarity and calculated from the HUMANn3 metabolic pathway abundance profiles.

Patients metadata				
Intervention group	Patients number	Age	Sex	Date
Inulin Patients	1	44	M	Tuesday 17 May 2016
Inulin Patients	4	49	F	Tuesday 17 May 2016
Inulin Patients	8	53	F	Tuesday 17 May 2016
Inulin Patients	10	52	F	Tuesday 17 May 2016
Inulin Patients	15	60	F	Tuesday 17 May 2016
Inulin Patients	18	46	F	Tuesday 17 May 2016
Inulin Patients	20	56	F	Tuesday 17 May 2016
Inulin Patients	22	45	F	Tuesday 17 May 2016
Inulin Patients	25	57	F	Tuesday 17 May 2016
Inulin Patients	30	37	F	Tuesday 17 May 2016
Kefir Patients	3	20	M	Tuesday 17 May 2016
Kefir Patients	6	35	F	Tuesday 17 May 2016
Kefir Patients	7	51	F	Wednesday 18 May 2016
Kefir Patients	12	42	F	Tuesday 17 May 2016
Kefir Patients	13	57	M	Tuesday 17 May 2016
Kefir Patients	16	40	M	Tuesday 17 May 2016
Kefir Patients	19	50	F	Tuesday 17 May 2016
Kefir Patients	23	31	M	Tuesday 17 May 2016
Kefir Patients	27	25	F	Tuesday 17 May 2016
Kefir Patients	28	31	F	Tuesday 17 May 2016
Yakult patients	2	28	M	Wednesday 18 May 2016
Yakult patients	5	40	M	Tuesday 17 May 2016
Yakult patients	9	42	M	Tuesday 17 May 2016
Yakult patients	11	51	M	Tuesday 17 May 2016
Yakult patients	14	57	F	Tuesday 17 May 2016
Yakult patients	17	29	F	Tuesday 17 May 2016
Yakult patients	21	60	F	Tuesday 17 May 2016
Yakult patients	24	51	F	Tuesday 17 May 2016
Yakult patients	26	58	F	Tuesday 17 May 2016
Yakult patients	29	36	F	Tuesday 17 May 2016

Observations

Bristol Stool	Fat %	Weight(kg)	Sys BP	Dia BP	Heart Rate	Height (cm)
4	12.0	63.8	125	68	62	171
4	41.8	87.6	144	94	65	168
3	30.9	67.8	110	71	63	168
4	23.9	71.2	110	78	66	174
5	44.4	83.3	124	93	96	167
4	31.5	68.1	111	69	62	161
3	38.8	78.8	116	71	61	171
Not answered	28.7	65.9	115	71	56	167
4	45.2	83.2	138	92	84	163
3	34.5	76.2	113	66	86	173
4	15.0	78.7	113	58	56	184
3	30.1	67.2	109	61	67	163
4	39.3	76.0	142	83	71	164
4	46.9	104.1	146	94	72	179
4	16.2	68.9	152	102	86	179
4	19.7	93.8	122	69	57	191
3	27.0	66.2	107	71	67	166
3	10.9	82.2	131	77	60	191
Not answered	24.1	58.8	114	67	80	160
3	13.6	52.8	126	74	82	178
3	16.9	69.6	120	76	70	170
4	15.2	73.1	138	83	66	177
4	32.6	112.3	130	89	78	191
4	34.0	110.8	143	89	75	186
3	34.7	67.9	139	88	79	168
4	25.4	56.2	108	70	74	155
4	28.2	57.9	125	65	75	160
2	39.9	84.5	135	98	69	156
4	21.5	50.5	119	74	58	153
5	31.7	66.9	130	73	63	163

	EQ-5D-5L				
BMI(printout)	Mobility(1-5)	Self-Care(1-5)	Usual(1-5)	Pain(1-5)	Anx/Dep(1-5)
21.8	1	1	1	1	1
31.0	1	1	1	2	1
24.0	1	1	1	1	1
23.5	1	1	1	2	1
29.9	1	1	1	1	1
26.3	1	1	1	1	1
26.9	1	1	1	1	1
23.6	1	1	1	1	1
31.3	1	1	1	2	2
25.5	1	1	1	1	1
23.2	1	1	1	1	1
25.3	1	1	1	1	1
28.3	1	1	1	1	1
32.5	1	1	1	1	1
21.5	1	1	1	1	1
25.7	1	1	1	1	1
24.0	1	1	1	1	1
22.5	1	1	1	1	1
23.0	1	1	1	1	1
16.7	1	1	1	1	1
24.1	1	1	1	1	1
23.3	1	1	1	1	1
30.8	1	1	1	1	1
32.0	1	1	1	1	1
24.1	1	1	1	1	1
23.4	1	1	1	1	1
22.6	1	1	1	1	2
34.7	1	1	1	3	1
21.6	1	1	1	1	1
25.2	1	1	1	1	2

Health(0-100)	Continuous(1-7)	After eat(1-7)	Fasting(1-7)	Doesn't decline(1-7)
80	1	1	1	1
90	1	1	1	1
80	1	1	1	1
70	1	1	1	1
80	1	1	1	1
85	1	2	1	Not answered
90	1	1	1	1
90	1	1	1	1
80	1	1	1	1
90	1	1	1	1
90	1	1	1	1
75	1	3	1	3
85	1	1	1	1
90	1	Not answered	Not answered	Not answered
85	1	1	1	1
85	1	1	1	Not answered
90	1	1	1	1
85	1	1	1	1
100	1	1	1	1
95	1	1	1	1
100	1	1	1	1
95	1	1	1	1
60	1	1	1	1
90	1	1	1	1
100	1	1	1	Not answered
81	2	1	1	1
91	1	1	1	1
75	1	1	1	1
90	1	1	Not answered	Not answered
90	1	1	1	1

Heartburn(1-7)	Regurg(1-7)	Abdo rumbling(1-7)	Bloated(1-7)	Empty(1-7)	Nausea(1-7)
1	1	2	1	1	2
1	1	2	2	1	1
2	1	2	1	1	2
1	1	1	1	1	1
2	1	1	1	1	1
1	1	2	2	2	1
1	1	2	1	2	1
1	1	2	1	2	1
3	1	1	1	1	1
2	1	2	1	1	2
1	1	1	1	1	1
1	1	2	4	1	1
1	1	2	2	2	1
1	1	1	1	1	1
1	1	2	1	2	1
1	1	3	1	1	1
1	1	1	1	1	1
1	1	2	1	1	1
1	1	1	1	1	1
1	1	2	1	2	1
1	1	4	1	1	1
1	1	2	1	2	1
1	1	1	1	1	1
2	1	2	1	1	1
1	1	1	1	1	1
1	1	2	1	2	1
1	1	2	1	2	1
2	2	3	3	3	1
1	1	1	1	1	1
1	1	2	2	Not answered	1

Vomiting(1-7)	Loss Appetite(1-7)	Fullness(1-7)	Belching(1-7)	Flatulence(1-7)
1	2	2	2	3
1	1	1	2	2
1	1	2	1	2
1	1	1	1	2
1	1	1	2	1
1	1	2	1	2
1	1	2	1	2
1	1	2	2	2
Not answered	1	1	2	2
1	1	4	3	3
1	1	3	1	2
1	1	4	1	4
1	1	2	1	2
1	1	Not answered	1	1
1	1	1	2	2
1	1	1	1	3
1	1	2	2	2
1	1	2	1	2
1	1	1	1	1
1	1	1	2	2
1	1	2	1	4
1	1	3	2	2
1	1	1	1	1
1	1	2	2	2
1	1	2	2	2
1	1	2	1	3
1	1	2	1	2
1	1	1	2	4
1	1	2	1	1
1	1	3	3	2

Abdo symptoms

Vomit blood(1-7)	Diff swall liq(1-7)	Diff swall solid(1-7)	Black stools(1-7)	Bloody stools(1-7)
1	1	1	1	1
1	1	1	Not answered	Not answered
1	1	1	1	1
1	1	1	1	1
1	1	1	1	1
1	1	1	1	1
1	1	1	1	1
1	1	1	1	1
1	1	1	1	1
1	1	1	1	1
1	1	1	1	1
1	1	1	1	1
1	1	1	1	1
1	1	Not answered	1	Not answered
1	1	1	1	1
1	1	1	1	1
1	1	1	Not answered	Not answered
1	1	1	3	1
1	Not answered	Not answered	1	1
1	1	1	1	1
1	1	1	1	1
1	1	1	2	1
1	1	1	1	1
1	1	1	1	1
1	1	1	1	1
1	1	1	1	1
1	1	1	1	1
1	1	1	1	1
1	1	Not answered	Not answered	Not answered
1	1	1	1	1

with Mucus(1-7)	Hard stools(1-7)	Diarrhoea(1-7)	alt solid/loose(1-7)
1	1	2	2
Not answered	Not answered	Not answered	1
1	2	1	1
1	1	1	1
1	1	1	2
1	1	1	1
1	1	1	1
1	1	1	1
1	1	1	1
1	1	1	4
1	2	1	2
1	3	1	2
1	2	1	1
Not answered	Not answered	Not answered	Not answered
1	1	1	1
1	1	1	1
Not answered	Not answered	Not answered	1
1	1	1	1
1	1	1	1
1	1	1	1
1	1	1	1
2	3	2	2
1	1	1	1
1	1	2	1
1	1	1	1
1	2	1	2
1	1	2	2
1	4	1	2
Not answered	Not answered	Not answered	Not answered
1	1	1	1

constipation(1-7)	freq with pain(1-7)	Urgency(1-7)	Incomplete(1-7)	Pain (0-12.3cm)
1	1	1	1	0.65
Not answered	Not answered	Not answered	Not answered	2.75
2	1	1	1	0.75
1	1	3	1	0.65
1	1	1	1	0.00
1	1	1	1	0.80
1	1	1	1	0.30
1	1	2	1	0.35
1	1	2	2	0.30
1	1	1	1	1.00
1	1	1	1	0.00
2	2	1	1	3.20
1	1	1	1	0.35
Not answered	Not answered	Not answered	Not answered	0.00
1	1	1	2	0.30
1	1	1	1	0.00
Not answered	Not answered	Not answered	Not answered	0.00
1	1	1	2	0.50
1	1	1	1	0.00
1	1	1	1	0.00
1	1	1	1	0.00
1	1	1	1	0.00
1	1	1	1	0.00
1	1	1	1	0.30
1	1	1	1	0.00
1	1	1	1	4.65
1	1	1	1	0.90
4	3	1	3	3.80
Not answered	Not answered	Not answered	Not answered	0.00
1	1	3	1	0.40

GAD-7				
Feel nervous...(0-3)	Not being...(0-3)	Worrying...(0-3)	Trouble...(0-3)	Being so...(0-3)
1	0	1	1	0
0	0	0	0	0
0	0	0	0	0
0	0	0	0	0
0	0	0	0	0
0	0	0	1	0
0	0	0	0	0
0	0	0	0	0
1	0	1	1	0
0	0	0	0	0
0	0	0	0	0
1	0	1	1	0
1	0	1	1	0
0	0	0	0	0
0	0	1	0	0
0	0	0	0	0
0	0	0	0	0
0	0	1	1	0
0	0	0	0	0
1	0	1	1	0
0	0	0	0	0
0	0	0	0	0
0	0	0	0	0
0	0	0	0	0
0	0	Not answered	1	0
0	0	0	0	0
0	0	1	1	0
1	0	0	1	0
0	0	0	0	0
2	2	2	3	1

Becoming easily..(0-3)	Feeling afraid...(0-3)	Date	Bristol Stool
1	0	Monday 13 June 2016	4
1	0	Tuesday 14 June 2016	4
1	0	Monday 13 June 2016	3
0	0	Tuesday 14 June 2016	4
1	0	Tuesday 14 June 2016	5
1	0	Tuesday 14 June 2016	4
0	0	Tuesday 14 June 2016	3
0	0	Tuesday 14 June 2016	4
0	0	Tuesday 14 June 2016	4
1	0	Monday 13 June 2016	4
0	0	Thursday 16 June 2016	5
0	0	Wednesday 15 June 2016	3
1	0	Monday 13 June 2016	4
0	1	Wednesday 15 June 2016	4
1	0	Monday 13 June 2016	4
0	0	Friday 17 June 2016	4
0	0	Wednesday 15 June 2016	3
0	1	Thursday 16 June 2016	3
0	0		
2	0	Wednesday 15 June 2016	3
0	0	Tuesday 14 June 2016	4
0	0	Monday 13 June 2016	4
0	0	Wednesday 15 June 2016	4
0	0	Wednesday 15 June 2016	4
1	0	Monday 13 June 2016	4
1	1	Monday 13 June 2016	2 to 3
1	1	Wednesday 15 June 2016	5
1	1	Monday 13 June 2016	1
0	0	Wednesday 8 June 2016	4
2	1	Wednesday 15 June 2016	5

Observations						
Fat %	Weight(kg)	Sys BP	Dia BP	Heart Rate	Height (cm)	BMI(printout)
12.1	62.3	122	66	57	171	21.3
42.1	85.3	139	93	62	168	30.2
31.6	66.4	116	73	76	168	23.5
27.4	70.6	110	76	61	174	23.3
43.4	83.6	143	96	91	167	30.0
31.1	68.9	113	69	67	161	26.6
40.3	79.0	137	85	57	171	27.0
29.3	65.8	103	64	61	167	23.6
45.4	83.1	130	78	99	163	31.3
	78.0	109	63	75	173	Not answered
15.1	78.8	128	62	61	184	23.3
27.0	66.0	100	56	62	163	24.8
39.0	76.7	125	78	84	164	28.5
45.3	104.4	140	87	61	179	32.6
16.6	70.1	158	89	78	179	21.9
21.9	94.3	129	71	54	191	25.8
29.1	65.5	113	65	70	166	23.8
12.6	82.8	122	71	66	191	22.7
14.6	53.6	123	83	80	178	16.9
16.2	68.9	131	75	63	170	23.8
15.2	73.5	131	81	58	177	23.5
32.0	115.8	140	84	70	191	31.7
33.3	110.9	157	85	64	186	32.1
35.8	68.7	132	84	81	168	24.3
26.2	56.4	109	60	94	155	23.5
27.6	58.1	115	69	74	160	22.7
40.4	84.5	149	96	70	156	34.7
24.0	50.3	113	72	64	153	43.0
41.6	67.4	124	69	60	163	25.4

EQ-5D-5L					
Mobility(1-5)	Self-Care(1-5)	Usual(1-5)	Pain(1-5)	Anx/Dep(1-5)	Health(0-100)
1	1	1	1	1	75
1	1	1	2	1	90
1	1	1	1	2	70
1	1	1	1	1	75
1	1	1	1	1	80
1	1	1	1	1	80
1	1	1	1	1	95
2	1	1	2	1	85
1	1	1	1	1	85
1	1	1	1	1	80
Not answered	Not answered	Not answered	Not answe	Not answered	50
1	1	1	1	1	80
1	1	1	1	1	95
1	1	1	1	1	90
1	1	1	1	1	95
1	1	1	1	1	79
1	1	1	1	1	100
1	1	1	2	1	79
1	1	1	1	1	95
1	1	1	1	1	98
1	1	1	2	1	95
1	1	1	1	1	80
1	1	1	2	1	90
1	1	1	1	1	100
1	1	1	1	1	90
1	1	1	1	1	98
1	1	1	2	1	80
1	1	1	1	1	90
1	1	1	1	1	90

Continuous(1-7)	After eat(1-7)	Fasting(1-7)	Doesn't decline(1-7)	Heartburn(1-7)
1	Not answered	1	1	1
1	2	1	Not answered	1
1	1	1	1	1
1	1	1	1	1
1	1	1	Not answered	2
1	2	3	1	1
2	2	2	1	1
1	1	1	1	1
1	2	1	Not answered	1
1	3	1	1	4
1	2	1	1	3
1	2	2	1	1
1	1	1	Not answered	1
1	1	1	1	1
1	1	1	1	1
1	1	1	1	1
1	1	1	1	1
1	2	2	1	2
1	1	2	1	2
1	1	1	1	1
1	1	1	1	1
1	1	1	1	1
1	1	1	1	2
1	1	1	1	1
3	Not answered	Not answered	3	2
1	1	1	1	1
1	1	1	1	2
1	1	1	Not answered	1
1	4	1	4	2

Regurg(1-7)	Abdo rumbling(1-7)	Bloated(1-7)	Empty(1-7)	Nausea(1-7)
1	1	1	1	1
1	2	4	1	1
1	1	2	1	2
1	1	1	2	1
1	2	1	1	1
1	2	4	2	3
1	3	3	2	1
1	2	1	1	1
1	1	2	1	2
1	1	1	1	3
1	4	1	4	1
1	1	4	1	1
1	2	2	1	1
1	1	2	1	1
1	2	1	1	1
1	1	1	1	1
1	1	1	1	1
1	1	2	1	2
2	2	1	1	1
1	3	1	1	1
1	2	1	2	1
1	1	1	1	1
1	2	1	1	1
1	2	2	1	2
2	2	1	3	2
1	1	1	1	1
1	2	3	2	1
1	1	1	1	1
1	4	5	1	2

Abdo symptoms

Vomiting(1-7)	Loss Appetite(1-7)	Fullness(1-7)	Belching(1-7)	Flatulence(1-7)
1	1	Not answered	1	3
1	1	1	1	5
1	1	2	1	2
1	1	1	1	1
1	1	1	1	2
1	1	1	3	4
1	1	4	1	4
1	1	1	1	2
1	1	2	1	1
1	2	3	2	2
1	1	1	3	3
1	1	3	1	2
1	1	1	1	2
1	1	2	1	1
1	1	2	2	3
1	1	1	1	1
1	1	1	1	1
1	1	2	2	2
1	1	2	2	2
1	1	2	1	3
1	1	3	2	2
1	2	1	1	1
1	1	1	2	2
1	1	2	4	4
1	1	1	1	3
1	1	2	1	2
1	1	3	3	3
1	1	1	1	1
2	1	5	2	6

Vomit blood(1-7)	Diff swall liq(1-7)	Diff swall solid(1-7)	Black stools(1-7)	Bloody stools(1-7)
1	1	1	1	1
1	1	Not answered	1	1
1	1	1	1	1
1	1	1	1	1
1	1	1	1	1
1	1	1	1	1
1	1	1	1	1
1	1	1	1	1
1	1	Not answered	1	1
1	1	1	Not answered	Not answered
1	1	1	Not answered	Not answered
1	1	1	1	1
1	1	1	1	1
1	1	1	1	1
1	1	1	1	1
1	1	1	1	1
1	1	1	1	1
1	1	1	Not answered	1
1	1	1	1	1
1	1	1	1	1
1	1	1	1	1
1	1	1	1	1
1	1	1	1	1
1	1	1	1	1
1	1	1	1	1
1	1	1	1	1
1	1	1	1	1
1	1	1	Not answered	Not answered
1	1	1	1	2

with Mucus(1-7)	Hard stools(1-7)	Diarrhoea(1-7)	alt solid/loose(1-7)	constipation(1-7)
1	1	1	1	1
1	1	2	3	1
1	3	1	3	2
1	1	1	1	1
1	1	1	1	1
1	1	1	1	1
1	1	1	1	1
1	1	2	1	1
1	1	1	1	1
Not answered	Not answered	Not answered	1	Not answered
Not answered	Not answered	Not answered	Not answered	Not answered
1	3	1	1	3
1	1	1	1	1
1	1	1	1	1
1	1	1	1	1
1	1	1	1	1
1	1	1	1	1
1	2	1	1	2
1	1	1	1	1
1	1	1	1	1
2	2	1	2	1
1	1	1	1	1
1	1	2	1	1
1	1	1	1	1
2	2	1	2	1
1	1	1	2	1
1	4	1	2	4
Not answered	Not answered	Not answered	Not answered	Not answered
1	4	4	6	5

freq with pain(1-7)	Urgency(1-7)	Incomplete(1-7)	Pain (0-12.3cm)	Feel nervous...(0-3)
1	1	1	0.35	1
1	2	1	8.00	0
1	1	1	0.35	1
1	2	1	0.00	0
1	1	1	0.30	0
1	2	1	5.75	1
1	1	3	1.80	0
1	2	1	0.80	1
1	1	1	1.20	1
Not answered	Not answered	Not answered	2.80	1
Not answered	1	Not answered	6.00	0
Not answered	1	1	8.90	0
1	1	1	0.00	0
1	1	1	0.00	0
1	1	2	0.30	0
1	1	1	0.00	0
1	1	1	0.20	0
1	1	2	3.00	0
1	1	1	0.45	2
1	1	1	0.00	0
1	1	1	0.20	0
1	1	1	0.00	0
1	2	1	0.70	0
1	1	1	0.25	1
1	3	Not answered	4.35	0
1	1	1	0.35	1
3	1	3	2.70	0
Not answered	Not answered	Not answered	0.00	0
4	6	6	7.70	1

GAD-7				
Not being...(0-3)	Worrying...(0-3)	Trouble...(0-3)	Being so...(0-3)	Becoming easily..(0-3)
0	1	1	0	1
0	1	0	0	0
0	0	0	0	1
0	0	0	0	0
0	1	1	0	1
0	1	0	0	1
0	2	0	0	0
1	1	1	1	1
0	0	0	0	0
0	0	0	0	1
0	0	1	1	2
0	0	1	0	1
0	0	0	0	1
0	1	1	0	0
0	0	0	0	0
0	0	0	0	0
0	0	0	0	0
0	1	0	0	0
1	2	2	1	2
0	0	0	0	0
0	0	0	0	0
0	0	0	0	1
0	0	0	0	0
0	0	1	0	1
0	0	0	0	1
0	0	0	0	0
0	1	1	0	1
0	0	0	0	0
1	1	2	0	2

Feeling afraid...(0-3)
0
0
0
0
0
0
0
1
0
0
0
0
0
1
0
0
0
0
0
0
0
0
0
0
0
0
0
0
0

Supplementary table 1: Changes in clinical parameters of participants following FMP, Inulin and Kefir consumption.
