# Supplementary material for: Comparison of the relative impacts of acute consumption of an inulin-enriched diet, milk kefir or a commercial probiotic product on the human gut microbiome and metabolome
Source: NPJ Sci Food. 2023 Aug 16;7:41. doi: 10.1038/s41538-023-00216-z (PMC10432396; doi:10.1038/s41538-023-00216-z)
Supplement: Supplementary file 2 — Reporting Summary [file 41538_2023_216_MOESM2_ESM.pdf]

nature portfolio

Corresponding author(s): Paul Cotter

Last updated by author(s): May 10, 2023

Reporting Summary

Nature Portfolio wishes to improve the reproducibility of the work that we publish. This form provides structure for consistency and transparency in reporting. For further information on Nature Portfolio policies, see our Editorial Policies and the Editorial Policy Checklist.

Statistics

For all statistical analyses, confirm that the following items are present in the figure legend, table legend, main text, or Methods section.

n/a	Confirmed	
☐	☒	The exact sample size (*n*) for each experimental group/condition, given as a discrete number and unit of measurement
☐	☒	A statement on whether measurements were taken from distinct samples or whether the same sample was measured repeatedly
☐	☒	The statistical test(s) used AND whether they are one- or two-sided<br>*Only common tests should be described solely by name; describe more complex techniques in the Methods section.*
☐	☒	A description of all covariates tested
☐	☒	A description of any assumptions or corrections, such as tests of normality and adjustment for multiple comparisons
☐	☒	A full description of the statistical parameters including central tendency (e.g. means) or other basic estimates (e.g. regression coefficient) AND variation (e.g. standard deviation) or associated estimates of uncertainty (e.g. confidence intervals)
☐	☒	For null hypothesis testing, the test statistic (e.g. *F*, *t*, *r*) with confidence intervals, effect sizes, degrees of freedom and *P* value noted<br>*Give P values as exact values whenever suitable.*
☒	☐	For Bayesian analysis, information on the choice of priors and Markov chain Monte Carlo settings
☒	☐	For hierarchical and complex designs, identification of the appropriate level for tests and full reporting of outcomes
☐	☒	Estimates of effect sizes (e.g. Cohen's *d*, Pearson's *r*), indicating how they were calculated

Our web collection on statistics for biologists contains articles on many of the points above.

Software and code

Policy information about availability of computer code

Data collection	No Software was used
Data analysis	Raw whole-metagenome shotgun sequencing reads derived from faecal samples collected in this study and kefir samples generated by Walsh et al, were quality filtered and trimmed using a combination of Picard Tools v2.18.23 (https://github.com/broadinstitute/picard) and SAMtools v1.10. Kraken2 v 2.1.1 was used to determine the species-level microbial composition of the gut and kefir microbiome. Relative abundance of each species among samples was calculated using Bracken v2.2. To distinguish between false positive and present taxa, the Kraken2 script was performed using the parameter -report-minimizer-data. Identifications displaying a high distinct minimiser count but accounting for <1 % relative abundance were confirmed as true positive/negative results using the bioinformatics tool raspir v101 following the methodology outlined in. Microbial pathway analysis was performed using HUMAnN3 v3.1.1. The pan- genome of Lactococcus raffinolactis (Lc. raffinolactis) was constructed by Roary v3.13, using complete reference genomes available in NCBI [41]. Kefir-derived metagenome assembled genomes of the species Lc. raffinolactis were acquired from. Meta(genomes) were clustered using dREP v3.2.0. Meta(genome) annotation was performed using DRAM v1.2 and Prokka v1.14.

For manuscripts utilizing custom algorithms or software that are central to the research but not yet described in published literature, software must be made available to editors and reviewers. We strongly encourage code deposition in a community repository (e.g. GitHub). See the Nature Portfolio guidelines for submitting code & software for further information.

Data

Policy information about availability of data

All manuscripts must include a data availability statement. This statement should provide the following information, where applicable:

- Accession codes, unique identifiers, or web links for publicly available datasets
- A description of any restrictions on data availability
- For clinical datasets or third party data, please ensure that the statement adheres to our policy

Sequence data has been deposited in the European Nucleotide Archive (ENA) under the project accession number PRJEB26842.

Research involving human participants, their data, or biological material

Policy information about studies with human participants or human data. See also policy information about sex, gender (identity/presentation), and sexual orientation and race, ethnicity and racism.

Reporting on sex and gender	Sex is the only terminology used in this study. Sex information was given by the participants through written consent and available in the supplementary information. The study design included both Male and Female participants in as close to a 50%-50% ratio as was obtainable. A reported sex based analysis was not performed, reason limited sample size.
Reporting on race, ethnicity, or other socially relevant groupings	We recruited 29 healthy volunteers, aged from 18 to 65, for this study. Volunteers were members of the public and were enrolled from the Centre for Health Science Inverness (https://www.nes.scot.nhs.uk/contact-us/centre-for-health-science-inverness/). Volunteers provided written informed consent to take part in the study. Volunteers had not consumed probiotic-containing products, including commercial probiotic drinks or probiotic supplements, within the 6 weeks preceding the study. They had not taken antibiotics, antacids or proton pump inhibitors within the 2 weeks preceding the study. Volunteers did not have any of the following medical conditions: inflammatory bowel disease (IBD), irritable bowel syndrome (IBS), celiac disease, food allergies, gastroenteritis (within the 4 weeks preceding the study), heart valve abnormalities, prior rheumatic fever, diabetes or any other immune disorder. Volunteers did not follow any other dietary or lifestyle recommendations during this intervention. Subjects were controlled weekly via phone call for possible side effects and compliance in achieving the dietary requirements.
Population characteristics	See above
Recruitment	We recruited 29 healthy volunteers, aged from 18 to 65, for this study. Volunteers were members of the public and were enrolled from the Centre for Health Science Inverness (https://www.nes.scot.nhs.uk/contact-us/centre-for-health-science-inverness/). Volunteers provided written informed consent to take part in the study
Ethics oversight	None

Note that full information on the approval of the study protocol must also be provided in the manuscript.

Field-specific reporting

Please select the one below that is the best fit for your research. If you are not sure, read the appropriate sections before making your selection.

☐ Life sciences ☐ Behavioural & social sciences ☒ Ecological, evolutionary & environmental sciences

For a reference copy of the document with all sections, see nature.com/documents/nr-reporting-summary-flat.pdf

Ecological, evolutionary & environmental sciences study design

All studies must disclose on these points even when the disclosure is negative.

Study description	In the present study, we use metagenomic sequencing and metabolomics to identify changes in the human gut microbiota and metabolome of healthy participants following daily consumption, over 28 days, of either a single portion of a commercial FMP containing the probiotic L. casei, a traditional fermented milk beverage kefir or a diet enriched with the prebiotic inulin.
Research sample	Human feacal samples
Sampling strategy	Volunteers had not consumed probiotic-containing products, including commercial probiotic drinks or probiotic supplements, within the 6 weeks preceding the study. They had not taken antibiotics, antacids or proton pump inhi.Stool and urine samples were collected on Day 0 and Day 28. bitors within the 2 weeks preceding the study. Volunteers did not have any of the following medical conditions: inflammatory bowel disease (IBD), irritable bowel syndrome (IBS), celiac disease, food allergies, gastroenteritis (within the 4 weeks preceding the study), heart valve abnormalities, prior rheumatic fever, diabetes or any other immune disorder. Volunteers did not follow any other dietary or lifestyle recommendations during this intervention. After a 7 day run-in period (Day -7 to Day 0), volunteers consumed one of the following treatments daily over a 28 day intervention period (Day 0 to Day 28): 7 g of inulin (n=10), 247ml of traditional kefir (n=9), and 65 ml of a commercial probiotic (L. casei Shirota)-containing dairy beverage (n=10). The inulin group was asked to consume additional portions of foods such as vegetables and fruits that constituted part of their normal diet and

contained fibre, which amounted to 7 grams of natural inulin. 7 grams of natural inulin were selected based on the GI tolerance [29], as short- and long-term consumptions of inulin, given at a daily dose containing at least 5 g of inulin, is reported to be well tolerated by healthy subjects [30]. The kefir group were asked to consume a kefir milk product, produced by the inoculation of milk with a kefir grain. Kefir samples were kindly provided by Nourish Kefir (https://www.nourishkefir.co.uk/buy-kefir-here/). Lastly, the probiotic group where asked to consume a commercially available probiotic yoghurt style drink, containing live cultures of L. casei Shirota

Data collection	Stool and urine samples were collected on Day 0 and Day 28. Several clinical parameters were measured before and after treatment, including height, weight, and body fat. Anxiety was assessed using the GAD-7 questionnaire, while quality of life was assessed using the EQ-5D questionnaire. Abdominal symptoms regarding bloating, flatulence and bowel habit were also recorded
Timing and spatial scale	After a 7 day run-in period (Day -7 to Day 0), volunteers consumed one of the following treatments daily over a 28 day intervention period (Day 0 to Day 28): 7 g of inulin (n=10), 247ml of traditional kefir (n=9), and 65 ml of a commercial probiotic (L. casei Shirota)-containing dairy beverage (n=10).
Data exclusions	No Data was excluded from the analysis
Reproducibility	Dataset was made freely available. Custom scripts were constricted in R for reuasability
Randomization	Volunteers were randomly divided into groups
Blinding	Blinding was utilised in the Kefir and probotic groups

Did the study involve field work? ☒ Yes ☐ No

Field work, collection and transport

Field conditions	Hospital
Location	NHS highlands
Access & import/export	Several clinical parameters were measured before and after treatment, including height, weight, and body fat. Anxiety was assessed using the GAD-7 questionnaire, while quality of life was assessed using the EQ-5D questionnaire. Abdominal symptoms regarding bloating, flatulence and bowel habit were also recorded. Stool consistency was classified using the Bristol Stool Scale (BSS)
Disturbance	None

Reporting for specific materials, systems and methods

We require information from authors about some types of materials, experimental systems and methods used in many studies. Here, indicate whether each material, system or method listed is relevant to your study. If you are not sure if a list item applies to your research, read the appropriate section before selecting a response.

Materials & experimental systems

n/a	Involved in the study	
☒	☐	Antibodies
☒	☐	Eukaryotic cell lines
☒	☐	Palaeontology and archaeology
☒	☐	Animals and other organisms
☒	☐	Clinical data
☒	☐	Dual use research of concern
☒	☐	Plants

Methods

n/a	Involved in the study	
☒	☐	ChIP-seq
☒	☐	Flow cytometry
☒	☐	MRI-based neuroimaging
